# Oligometastatic Prostate Cancer: Molecular Imaging and Clinical Management Implications in the Era of Precision Oncology

**DOI:** 10.2967/jnumed.118.213470

**Published:** 2018-09

**Authors:** Hossein Jadvar

**Affiliations:** Division of Nuclear Medicine, Department of Radiology, University of Southern California, Los Angeles, California

The underlying biology of prostate cancer is complex and evolves from initial tumorigenesis to metastatic potentiation and castrate resistance. Metastases source directly from the primary tumor site but also can occur from an established metastatic site to another new metastatic site or even to the surgical bed, leading to local recurrence (1,2). This cross-metastatic site seeding is often associated with heterogeneous tumor clones with varying degrees of aggressiveness and resistance to therapy. The spatiotemporal clonal diversity of metastases suggest that not all metastases are created clinically equal and that targeted treatment of some key metastases, if clearly identifiable, can potentially improve systemic control of cancer and overall outcome, even in the setting of occult micrometastases.

Hellman and Weichselbaum proposed the existence of a clinical state that they termed *oligometastatic disease* as an intermediate step in cancer progression from a localized confined process to a disseminated state (spectrum theory of cancer) (3,4). The oligometastatic state is associated with a limited number of detectable metastases (variably defined as 1–3, 4, or 5 sites) with distinct presumably less aggressive biology (immature metastatic competence) in comparison to widespread polymetastatic disease (5,6). The postulate here is that oligometastatic lesions are early along their evolutionary line of metastatic potentiation. The management implication then follows that in some patients with oligometastases, cure may still be possible with definitive metastasis-directed therapies (MDTs) and minimal systemic toxic effects (7–14). Also the oligometastatic colony is removed or destroyed before it can evolve into more aggressive phenotype with untoward consequences locally and potential for facilitating tumor seeding of other sites.

Diagnosis of oligometastatic disease relies fundamentally on the performance of diagnostic imaging tools, which are advancing rapidly (15–18). The advent of more sensitive imaging technologies and availability of safe and effective localized non- or minimally invasive treatment options (e.g., stereotactic body radiation therapy [SBRT], local ablation, or surgical techniques) in the era of precision and personalized cancer care have led to increasing incidence and clinical interest in oligometastatic disease. The case for treating patients with limited metastatic disease is not a recent phenomenon; however, as Reyes and Pienta point out, the beneficial evidence for treatment of oligometastases in several cancers (e.g., breast, lung, colorectal, kidney, melanoma, and sarcoma) is overall weak (4).

Few studies have focused on treatment and clinical outcomes of oligometastatic prostate cancer. Azzam et al. used SBRT to treat up to 4 metastases in patients with recurrent prostate cancer after prior definitive treatment (19). Median survival was significantly longer in patients with treated oligometastatic disease than in those with treated polymetastatic disease (>3 vs. 11 mo, *P* = 0.02). More recently, Ost et al. reported on the results of a prospective, randomized, multicenter phase II trial comparing surveillance with MDT for oligometastatic prostate cancer recurrence (STOMP trial, NCT01558427) (20). Sixty-two asymptomatic noncastrate men with prior definitive treatment for primary prostate cancer who presented with biochemical recurrence with 3 or fewer extracranial metastases on choline PET were randomized (balanced on the basis of prostate-specific antigen doubling time and nodal vs. nonnodal metastases) to either surveillance or MDT (surgery or SBRT) of oligometastases. The primary endpoint was androgen-deprivation therapy–free survival with a median follow-up time of 3 y. The androgen-deprivation therapy–free survival was significantly longer with MDT than with surveillance alone (21 vs. 12 mo; hazard ratio, 0.55; log-rank *P* = 0.08, per-protocol analysis). Interestingly, 35% of patients undergoing surveillance experienced relatively short-duration spontaneous prostate-specific antigen declines without any therapy, whereas 30% of patients treated with MDT progressed to polymetastatic disease within the first year. A similar phase II trial (ORIOLE) is also under way, which randomizes men with noncastrate oligometastatic prostate cancer (identified on prostate-specific membrane antigen ligand imaging with ^18^F-DCFPyl PET/CT) to either surveillance or selective ablative radiotherapy of metastatic lesions, with the primary clinical endpoint of progression at 6 mo from randomization (NCT02680587) (21). Safety, efficacy, and effects of selective ablative radiotherapy–directed therapy of oligometastases on circulating tumor cells, circulating tumor DNA, and immunologic response will also be measured. Nevertheless, it still remains to determine whether the strategy of MDT-directed therapy of oligometastases (with or without additional systemic therapy to combat invisible micrometastases) leads to improved disease-specific or overall survival (22).

In view of data cited in the studies above, several issues remain to be settled before the concept of oligometastases can take potential hold in the routine clinical management of patients with prostate cancer. A biologic anchor is needed to decipher whether oligometastatic lesions are indeed different from polymetastatic lesions and how oligo lesions evolve to poly lesions (23). Some work has shown that oligometastases have micro-RNA expression profiles different from those of polymetastases (5). Additional studies that fully characterize the differential genotype and phenotype will not only advance our basic understanding of cancer evolution but also can be helpful in identifying those patients who most likely would benefit from MDT-directed therapy of oligometastases, thus elucidating whether we should “catch them all or not” as Murphy et al. elaborated (24). These additional studies can also provide opportunities for development of sophisticated interventions that may retain the biologic behavior of these few metastases as indolent, decreasing the risk for metastasis-to-metastasis seeding. There is need for a standardized definition of oligometastases preferably based on some biologic markers that can be tailored to a defined molecular imaging technique, rather than the current situation in which detection and localization of oligometastatic lesions are primarily dependent on the type and sensitivity of the diagnostic imaging that is used. Additionally, there is lack of uniformity in describing the oligometastatic condition as it is now used in various clinical scenarios with likely different underlying biology (25). These include synchronous oligometastases (with untreated primary cancer), metachronous disease (after definitive therapy of primary cancer), and induced oligometastases (when widespread disease is eradicated by systemic therapy but few drug shielded or resistant lesions remain).

Although the concept of oligometastasis is interesting, there is still more basic research that needs to be done to establish it firmly as a distinct biologic entity along the natural history of prostate cancer. The current unsystematic approach in detection and management of oligometastatic prostate cancer will need to be standardized so that future clinical trials can be designed appropriately and, more importantly, compared properly. These critical prerequisites will elucidate whether detection and treatment of oligometastases should be incorporated into the routine clinical management of patients with prostate cancer.

## DISCLOSURE

This study was supported by the National Institutes of Health grant numbers R21-EB017568 and P30-CA014089. No other potential conflict of interest relevant to this article was reported.
